# Polydatin prevent lung epithelial cell from *Carbapenem-resistant Klebsiella* *pneumoniae* injury by inhibiting biofilm formation and oxidative stress

**DOI:** 10.1038/s41598-023-44836-7

**Published:** 2023-10-18

**Authors:** Xiaodan Guan, Liang Jin, Huifen Zhou, Jing Chen, Haofang Wan, Yida Bao, Jiehong Yang, Daojun Yu, Haitong Wan

**Affiliations:** 1 Zhejiang Chinese Medical University, No. 548, Binwen Road, Binjiang District, Hangzhou, 310053 Zhejiang People’s Republic of China; 2 Hangzhou First People’s Hospital, Hangzhou, 310003 Zhejiang People’s Republic of China; 3 Shanghai University of Traditional Chinese Medicine, Shanghai, 201203 People’s Republic of China

**Keywords:** Cell biology, Microbiology, Physiology, Diseases

## Abstract

*Carbapenem-resistant Klebsiella pneumoniae* (CRKP) causes severe inflammation in various infectious diseases, such as bloodstream infections, respiratory and urinary tract infections, which leads to high mortality. Polydatin (PD), an active ingredient of Yinhuapinggan granule, has attracted worldwide attention for its powerful antioxidant, anti-inflammatory, antitumor, and antibacterial capacity. However, very little is known about the effect of PD on CRKP. In this research, we evaluated the inhibitory effects of PD on both the bacterial level and the bacterial-cell co-culture level on anti-biofilm and efflux pumps and the other was the inhibitory effect on apoptosis, reactive oxygen species (ROS), mitochondrial membrane potential (MMP) after CRKP induction. Additionally, we validated the mechanism of action by qRT-PCR and western blot in human lung epithelial cells. Firstly, PD was observed to have an inhibitory effect on the biofilm of CRKP and the efflux pump AcrAB-TolC. Mechanically, CRKP not only inhibited the activation of Nuclear Factor erythroid 2-Related Factor 2 (Nrf-2) but also increased the level of ROS in cells. These results showed that PD could inhibit ROS and activate Nrf-2 production. Together, our research demonstrated that PD inhibited bacterial biofilm formation and efflux pump AcrAB—TolC expression and inhibited CRKP-induced cell damage by regulating ROS and Nrf-2-regulated antioxidant pathways.

## Introduction

*Klebsiella pneumoniae* (KP), is a Gram-negative opportunistic pathogen, which often causes hospital-acquired infections, such as bloodstream infections, respiratory and urinary tract infections, and especially underlying diseases^1^. In addition, KP is becoming increasingly resistant to the most effective antibiotics, such as the prevalence of imipenem-resistant KP increased each year in China, from 3.0% in 2005 to 25.0% in 2018^2,3^. Owing to the overuse of antibiotics for decades, the emergence of *Carbapenem-resistant* *Klebsiella pneumoniae* (CRKP) poses a great challenge to the antibacterial treatment of clinical infection^4^. Among various resistance mechanisms, overexpression of the efflux pump is an important mechanism of KP resistance^5^. An important virulence characteristic of KP is the ability to form biofilms, which makes it difficult for conventional antimicrobial agents to penetrate; therefore, it repeatedly causes chronic bacterial behavior infections^6,7^. In recent years, biofilm and efflux pump are demonstrated to be a certain relationship, and several studies suggested that efflux pumps play at least four different roles in biofilm formation: efflux of extracellular polymeric substances (EPSs) and/or Quorum sensing (QS) and quorum quenching (QQ) molecules to facilitate biofilm matrix formation and regulate QS, respectively; indirect regulation of genes involved in biofilm formation; efflux of harmful molecules, such as antibiotics and metabolic intermediates; and influencing aggregation through promoting or preventing adhesion to surfaces and other cells^8^. Therefore, finding new drug candidates to treat bacterial infectious diseases and exploring their potential mechanisms is imperative.

**Figure 1:**
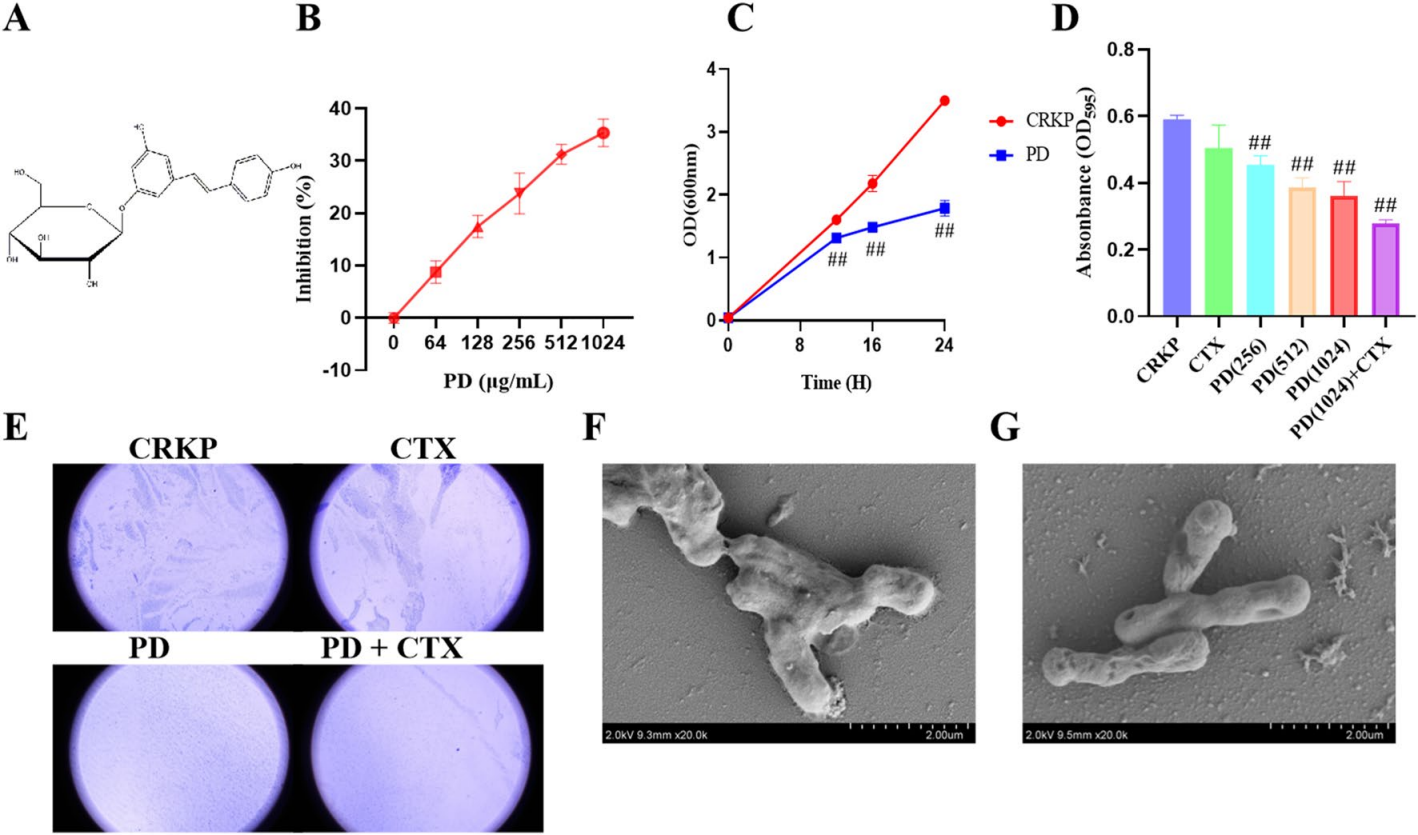
Characterization of CRKP biofilm. The structure of the polydatin (**A**). Growth curve analysis of bacterial growth (**B**) and time-killing curve of PD for CRKP (**C**). Inhibitory effects of PD on biofilm formation by CRKP were determined by Crystal violet assay (**D**). SEM images of CRKP (**F**). SEM images of CRKP + PD (**G**). CRKP morphological changes induced with or without the addition of PD were observed under an inverted microscope (**E**). ^#^*P* < 0.05 and ^##^*P* < 0.01 vs. the CRKP alone group.

Polydatin (PD, 3,4'-5-Trihydroxystilbene-3-β-D-glucopyranoside), a naturally occurring stilbene, owns a variety of health-promoting effects and powerful antioxidant, anti-inflammatory and anti-tumor effects (Fig. 1 A)^9–11^. PD is also an important component of traditional Chinese medicine compounds, Yinhuapinggan (YHPG) granules used to treat respiratory infections^12–16^. YHPG was composed of six herbs, which had the effect of clearing heat and detoxifying^17^. Investigations into the pharmacodynamics and mechanism of YHPG in animals and cells had demonstrated its antiviral, antibacterial, antipyretic, analgesic, cough relieving, and immunomodulatory effects^17^. In previous studies, we found that YHPG had a positive effect on the treatment of viral infectious respiratory diseases^14^. At the same time, recent studies had shown that oxidative stress played an important role in the pathogenesis of many inflammatory diseases and induced cell apoptosis and that high levels of reactive oxygen species produced by elements such as LPS and KP lead to oxidative stress^18–20^. PD had demonstrated to reduce inflammation and pulmonary fibrosis caused by Mycoplasma pneumoniae infection by blocking the NLRP3 inflammasome and NF-κB pathway^21^. In addition, research had demonstrated that PD could inhibit the generation of ROS and NF-κB p65 activation, suggesting that PD suppressed S. aureus lipoteichoic acid (LTA)-induced injury by attenuating ROS generation and TLR2-NFκB signalling^9^. However, it remained unclear whether PD possessed anti-microbial activity in bacterial pneumonia and its underlying mechanism of action.

Therefore, this study mainly investigated the bacteriostatic effect of PD extracts and their protective effects against CRKP-induced cell damage. The results indicated that PD had a certain inhibitory effect on the biofilm of CRKP and the efflux pump AcrAB-TolC. Mechanically, CRKP not only inhibited the activation of Nuclear Factor erythroid 2-Related Factor 2 (Nrf-2) but also increased the level of ROS in cells. Furthermore, we used Nrf-2 inhibitors to further investigate the role of the Nrf-2 signaling pathway in CRKP-induced cell damage. These results showed that PD could inhibit ROS and activate Nrf-2 production. These findings provided valuable insights into potential novel strategies for comprehensive treatment of bacterial pneumonia.

## Results

### Characteristics of CRKP biofilm

The bacterial growth curve concluded that the MIC value of PD for CRKP was greater than 1024 μg/mL, and PD showed antibacterial activity against CRKP strain at 1024 μg/mL (Fig. 1B,C). Biofilm formation abilities of CRKP detected by crystal violet staining were shown in Fig. 1D and Table 1, the results proved that 1024 μg/mL PD was effective in biofilm inhibition. This finding was similar to the results under an inverted microscope, PD had an inhibitory effect on the biofilm formation of CRKP, and combined with cefotaxime sodium (CTX) had an inhibitory effect on the biofilm formation of CRKP (Fig. 1E). Further, Scanning Electron Microscope (SEM) was used to observe the thallus shape of CRKP, and it was found that the thallus of CRKP without drug addiction was full and the surface did not collapse (Fig. 1F), but the group with PD was not full and its surface collapsed (Fig. 1G).

**Table 1:** Biofilm formation abilities of different treatment groups.

	CRKP	CTX	PD	PD + CTX
Biofilm formation capability (%)	100.00%	85.45%	61.01%	47.11%

### Relationship of biofilm formation and efflux pump in CRKP

**Figure 2:**
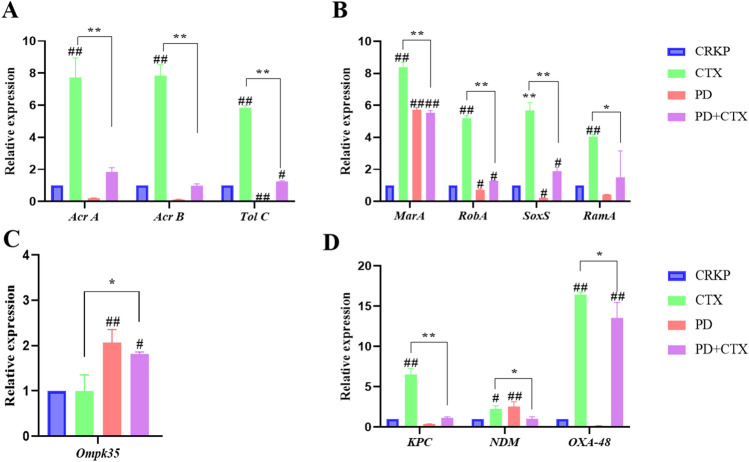
Effect of PD on the expression of PCR and proteins in CRKP. The mRNA expression of *Acr A, Acr B and Tol C* (**A**). *MarA, RobA*, *SoxS,* and *RamA* (**B**), *Ompk35* (**C**), *KPC, NDM, and OXA-48* (**D**) of CRKP infected cells were assessed by RT-PCR in the presence or absence of PD after 16 h incubation. ^#^*P* < 0.05 and ^##^*P* < 0.01 vs. the CRKP alone group. ^*^P < 0.05 and ^**^P < 0.01 vs. the CTX group.

Additionally, to elucidate the relationship between biofilm and efflux pump in CRKP, we further quantified them by qRT-PCR based on biofilm detection. The relative transcription levels of *AcrA*, *AcrB,* and *TolC* were significantly increased in the CTX group. However, after CTX was combined with PD, the transcript levels of *AcrA*, *AcrB,* and *TolC* were decreased compared with the same concentration of the CTX alone group, which was statistically significant (Fig. 2A).

*MarA* and *RamA* were stress regulators, which regulated the expression of AcrAB-TolC by adjusting their expression levels when the environment changed^22^. The relative transcription levels of *MarA*, *RobA,* and *SoxS* were significantly upregulated in the CTX group (Fig. 2B). Similarly, the expressions of *MarA*, *RobA,* and *SoxS* were significantly down-regulated after combined treatment with PD and CTX (compared with the CTX group) (Fig. 2B). *Ompk35* was a protein gene, and the expression of *Ompk35* could increase the sensitivity of CRKP to antibiotics. The relative transcription levels of *Ompk35* were significantly upregulated in the group exposed to PD compared with the CRKP group (Fig. 2C). Similarly, the results were identical for *KPC*, *NDM,* and *OXA-48*(Fig. 2D).

In conclusion, the addition of CRKP to CTX increased the expression of AcrAB-TolC, leading to the excretion of CTX, which reduced the bactericidal capacity of CTX. However, after CRKP was combined with PD, the expression of AcrAB-TolC was inhibited, thus reducing the excretion of drugs and toxins.

### Effect of PD on protein

**Figure 3:**
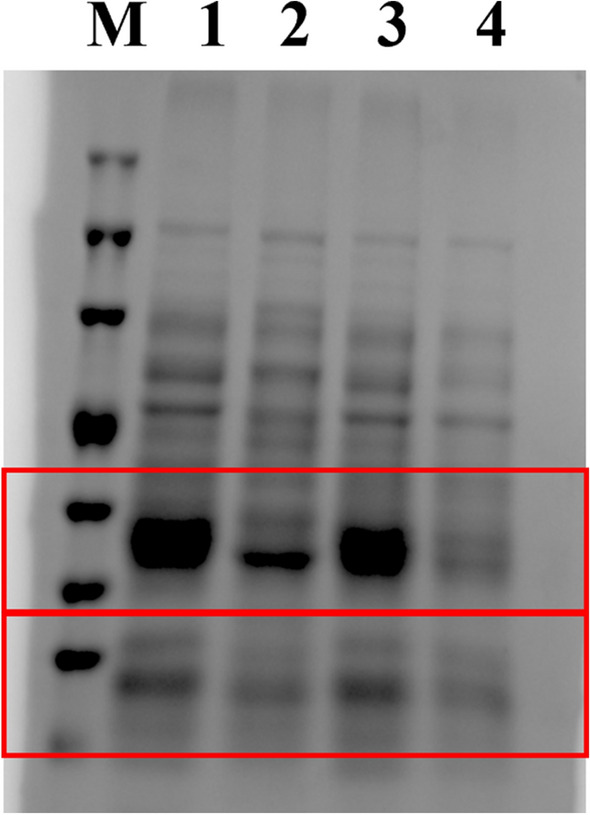
Effect of PD on proteins in CRKP. Total protein profile of SDS-PAGE cells. M: Molecular weight marker; 1: CRKP of the control group; 2: CRKP treated with CTX; 3: CRKP treated with PD; 4; CRKP treated with PD + CTX.

Herein, we determined the effect of PD on the protein of CRKP by SDS-PAGE. Many protein bands appeared significantly shallower compared to the CRKP group after CTX and CTX + PD treatment (Fig. 3).

### PD reduced cell damage caused by CRKP infection

**Figure 4:**
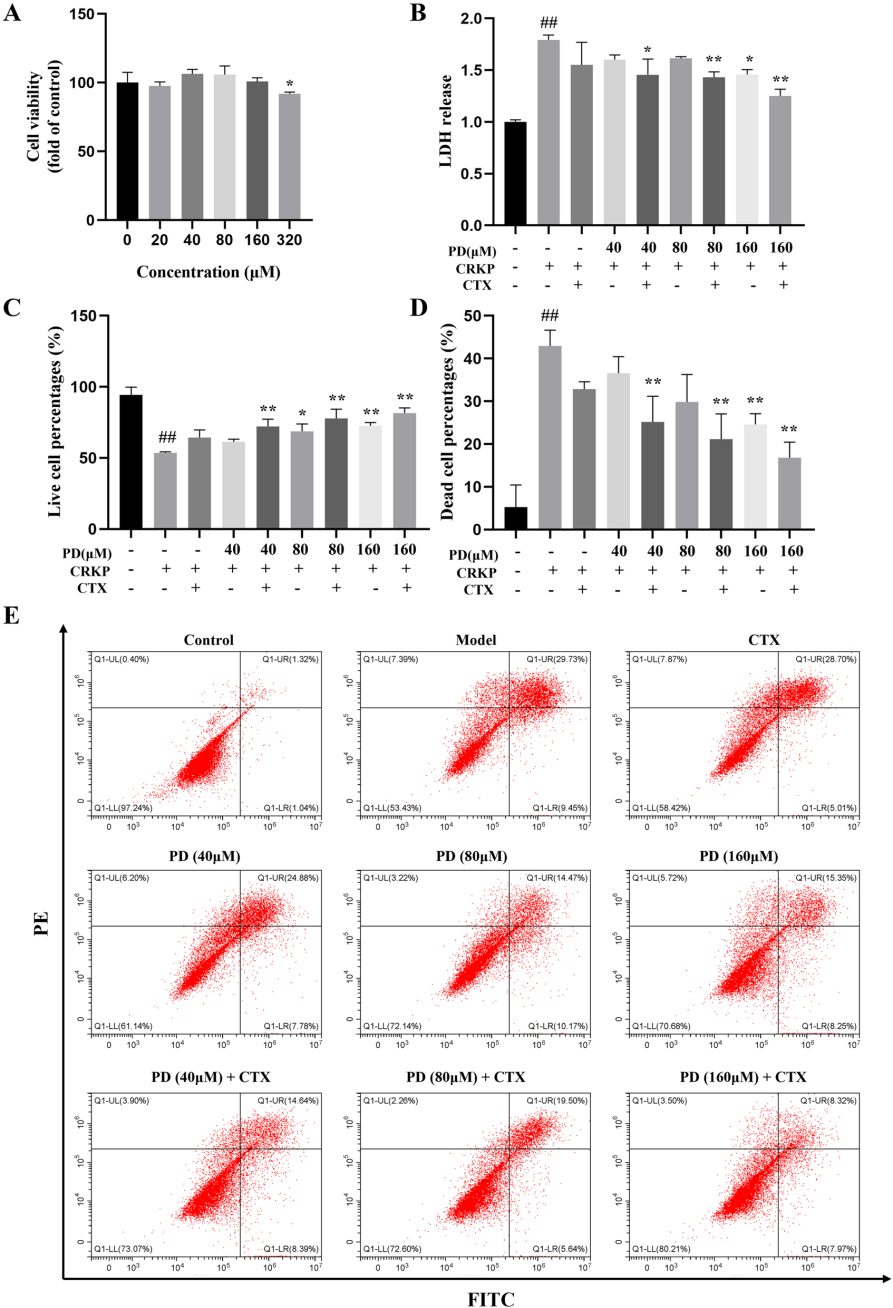
PD attenuated cellular apoptosis induced by CRKP. (**A**) CCK8 analyzed the cell viability after 24 h incubation with various concentrations of PD. CRKP-induced cellular injury induced by PD was detected by LDH (**B**). (**E**) A representative result for the changes in alive cell proportion and dead cell proportion was assessed by flow cytometry, and the statistical outcomes were shown (**C**-**D**). ^##^*P* < 0.01 vs. the control group; ^*^P < 0.05 and ^**^P < 0.01 vs. the CRKP alone group. Model: CRKP (MOI = 30) alone group.

To investigate the effect of PD on CRKP-infected A549 cells. This study initially evaluated the cytotoxicity of PD. The results proved no significant change in cell viability when PD-induced cell damage was less than 160 μM (62 μg/mL) (Fig. 4A). Therefore, a PD concentration of 40–160 μM was used in the follow-up experiment. To elucidate whether PD could protect cells from CRKP damage, we further researched the survival status of cells by Annexin V-FITC and PI. The analysis showed that PD reduced the proportion of dead cells induced by CRKP and increased the proportion of living cells, as well as showed a synergistic effect with CTX (Fig. 4C,E). In addition, we conducted an analysis and the LDH assay corroborated the results that PD functions with the same results obtained by flow cytometry (Fig. 4B).

### PD reduced the adhesion rate and invasion rate of CRKP

**Figure 5:**
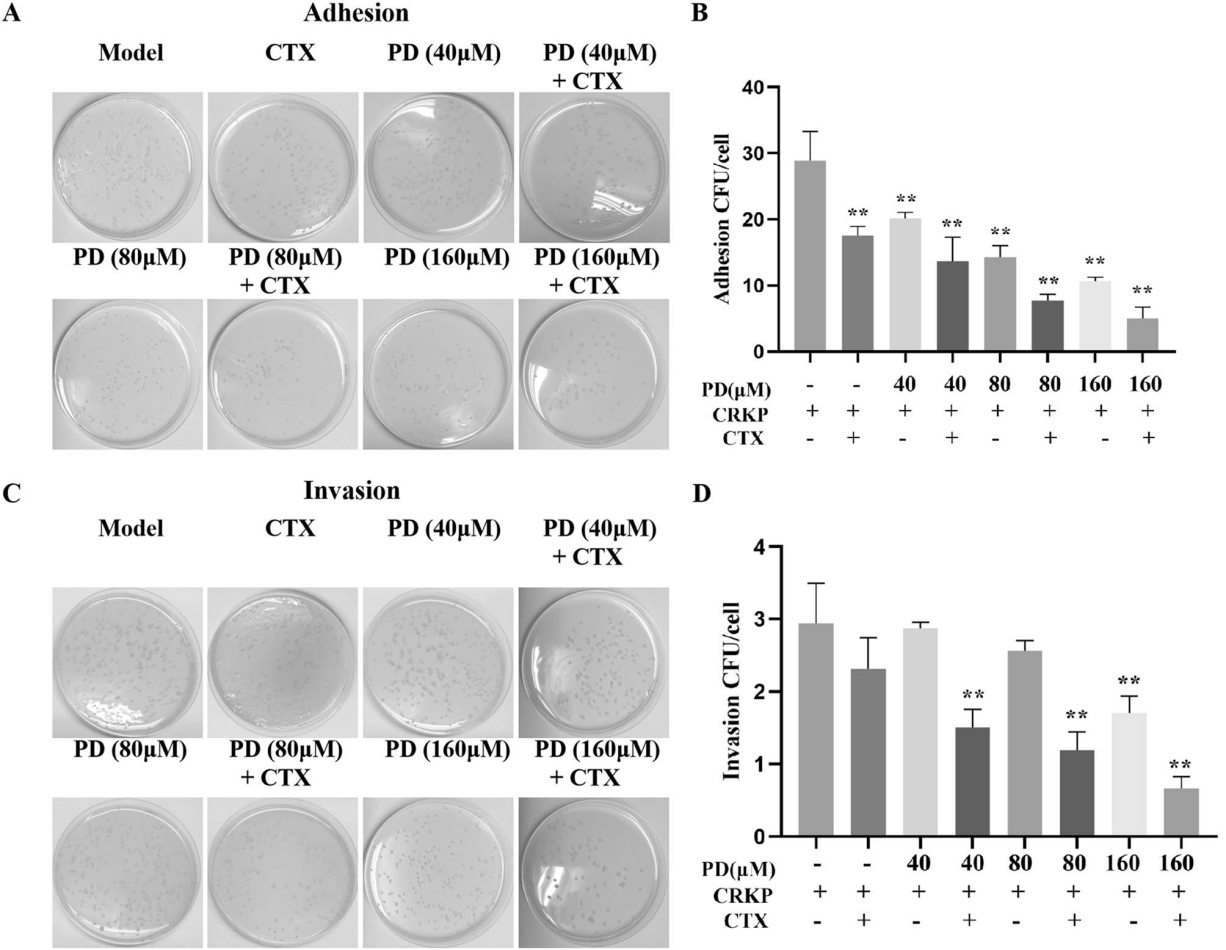
Effects of PD on the adhesion and invasion rate of CRKP-infected A549 cells. Cells were infected by CRKP with or without PD or CTX for 12 h, and then an appropriate amount of cell lysate was applied to LB solid medium for 24 h (**A**), and the statistical results were shown for adhesion (**B**). On the other hand, infected-cell were cultured in F12K complete medium containing antibiotics, and extracellular CRKP was washed with PBS. The appropriate amount of CRKP-infected A549 cell lysate was applied to LB solid medium for 24 h and counted (**C**), and the statistical results were shown for invasion (**D**). ^*^P < 0.05 and ^**^P < 0.01 vs. the CRKP alone group.

Next, we investigated the effect of PD on CRKP on cell adhesion and invasion. The results proved that the adhesion and invasion of CRKP to cells were observed to be rapid, while PD decreased the adhesion and invasion capacity of CRKP (Fig. 5A-D). In addition, compared with CTX alone, PD and CTX synergistically protected cells from CRKP adhesion and invasion. These results indicated that PD could inhibit adhesion and invasion of CRKP.

### PD decreased the production of proinflammatory cytokines induced by CRKP infection

**Figure 6:**
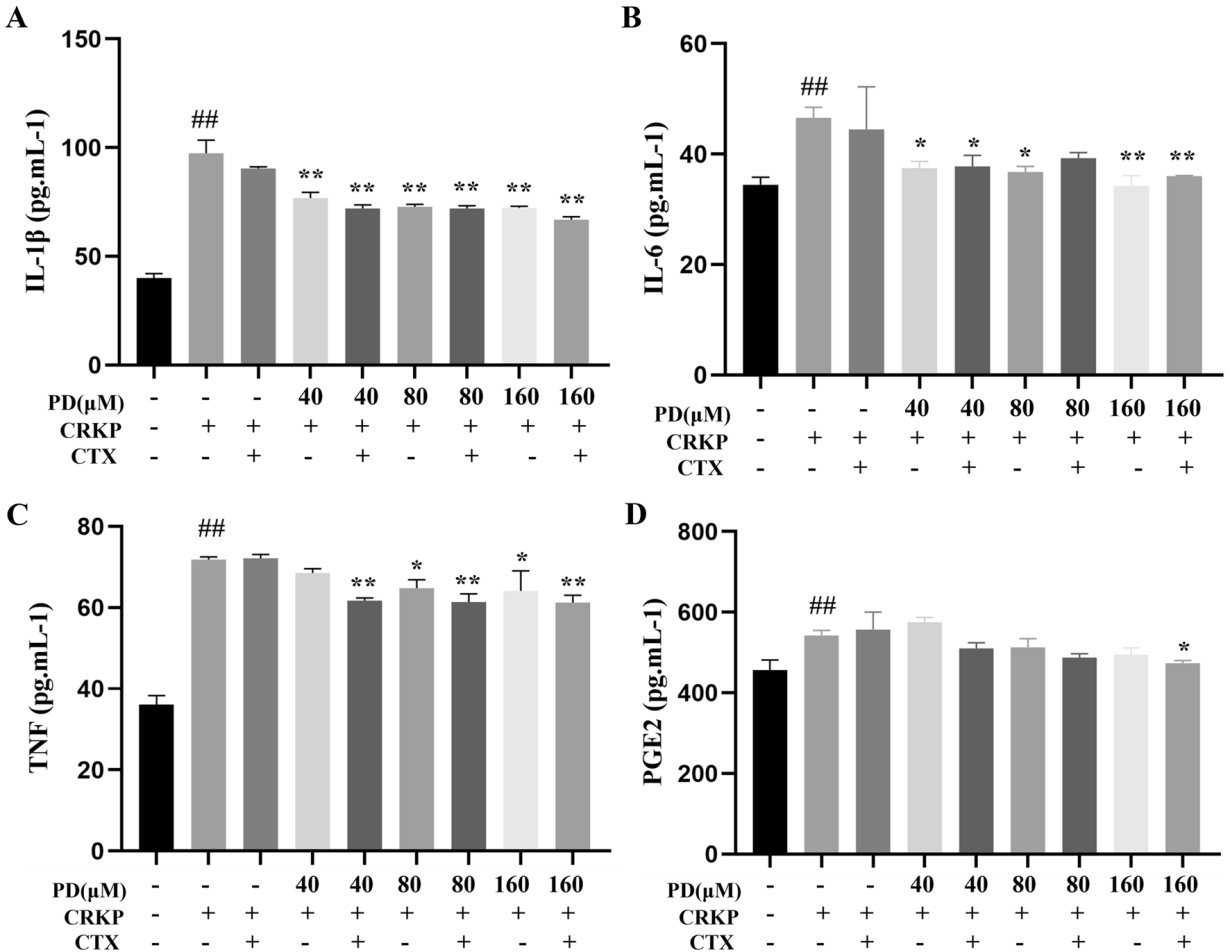
PD ameliorated the cellular inflammatory response brought on by CRKP stimulation. The production of IL-1β (**A**), IL-6 (**B**), TNF-α (**C**), and PGE2 (**D**) of CRKP-infected cells with or without PD or CTX in the supernatant were analyzed by ELISA. ^##^*P* < 0.01 vs. the control group; ^*^P < 0.05 and ^**^P < 0.01 vs. the CRKP alone group.

In this study, Enzyme-linked immunosorbent assay (ELISA) was performed to investigate the effect of PD on CRKP-infected proinflammatory cytokines. As shown in Fig. 6A-D, Interleukin-1β (IL-1β), Tumor necrosis factor-α (TNF-α), Interleukin-6 (IL-6), and Prostaglandin E2 (PGE2) concentrations were significantly higher in the CRKP group than in the control group, while the expression of these cytokines (except PGE2) was inhibited in the PD group. These results indicated that PD suppressed the CRKP-stimulated inflammatory expression.

### PD inhibited the downregulation of MMP in CRKP-induced cells by reducing ROS levels

**Figure 7:**
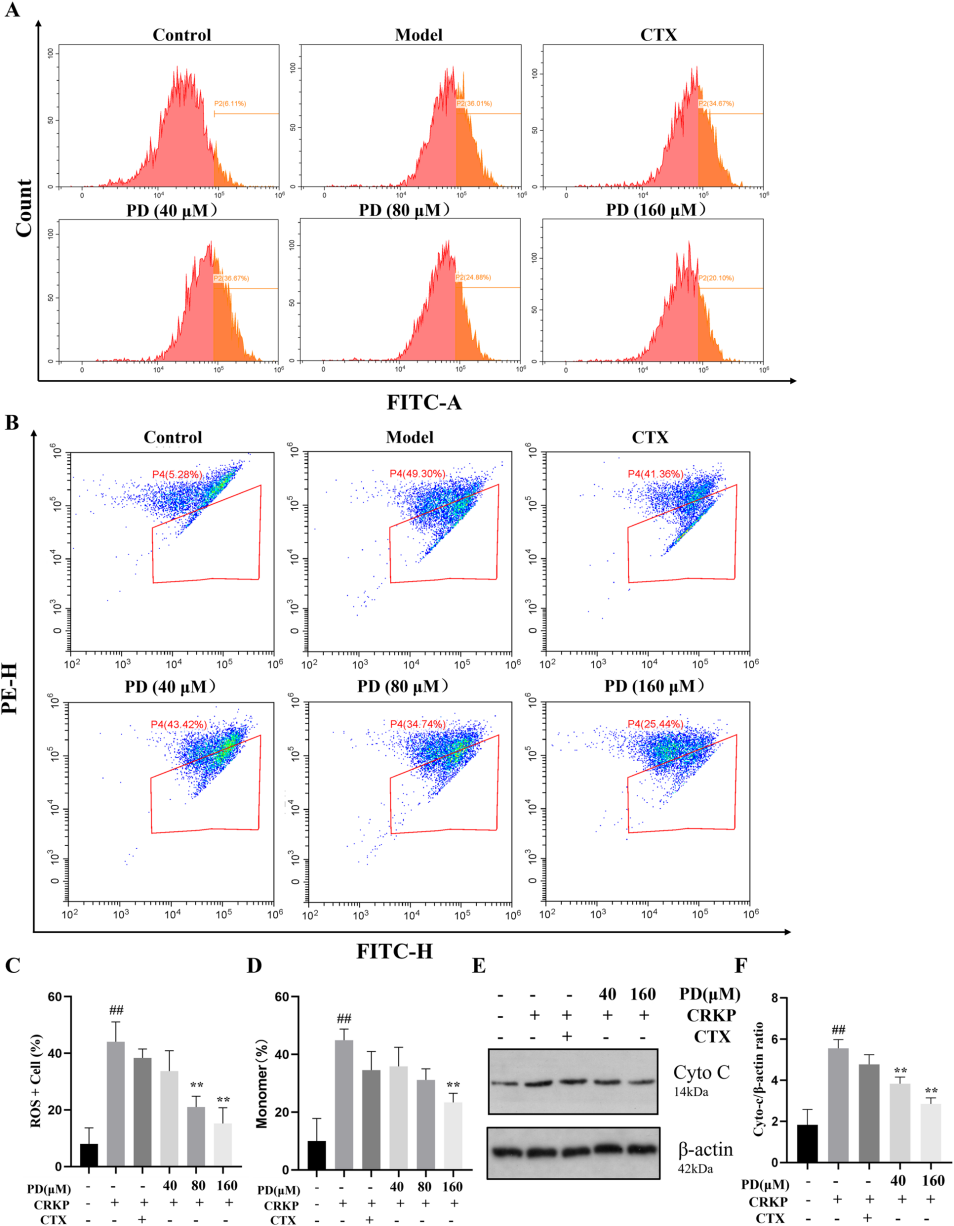
PD reduced mitochondrial dysfunction by inhibiting cellular damage induced by CRKP. A549 cell cultured with CRKP and with PD for 12 h was collected to evaluate intracellular ROS levels using a DCFH-DA probe. A representative flow cytometric result of ROS expression in various groups was analyzed (**A**), and a statistical graph was built (**C**). A549 cells incubated with CRKP for 12 h were collected for assessment of MMP using JC-1 staining (**B**, **D**). A representative result of the protein expression of Cyto-c among all groups was analyzed by western blot, and a statistical result was calculated (**E**–**F**). ^##^*P* < 0.01 vs. the control group; ^*^P < 0.05 and ^**^P < 0.01 vs. the CRKP alone group.

Oxidative stress may oxidize and destroy KP's biofilm, resulting in the loss of major membrane proteins and KP activity^23^. To confirm whether PD could modulate ROS expression induced by CRKP infection, we measured ROS concentration and found that CRKP responded significantly more to cell stimulation than the control group. Meanwhile, PD exhibited antioxidant effects, as demonstrated by a dose-dependent reduction in ROS generation (Fig. 7A,C). Next, we wondered whether the changes in the influence of CRKP infection on mitochondrial membrane potential (MMP) might be induced by ROS. Compared with the control group, flow cytometry results noted that MMP was significantly down-regulated after CRKP stimulation, while MMP down-regulated after PD treatment was improved (Fig. 7B and D). The above results indicated that PD suppressed CRKP-induced MMP downregulation in cells, thereby inhibiting the production of intracellular oxidative stress. In addition to MMP, Cyto-c was also an important component of the mitochondrial respiratory chain, which could activate the downstream cysteine-regulated apoptotic pathway. Therefore, we also analyzed the regulation of Cyto-c in PD by WB. As shown in Fig. 7E,F, the expression of Cyto-c in the CRKP group was higher than that in the control group. However, the high concentration of the PD group had an inhibitory effect on the expression of Cyto-c. These results confirmed that PD could reduce oxidative stress in CRKP infection and inhibit the corresponding downregulation of MMP.

### PD inhibited oxidative stress damage in CRKP-induced cells by activating the Nrf-2 pathway

**Figure 8:**
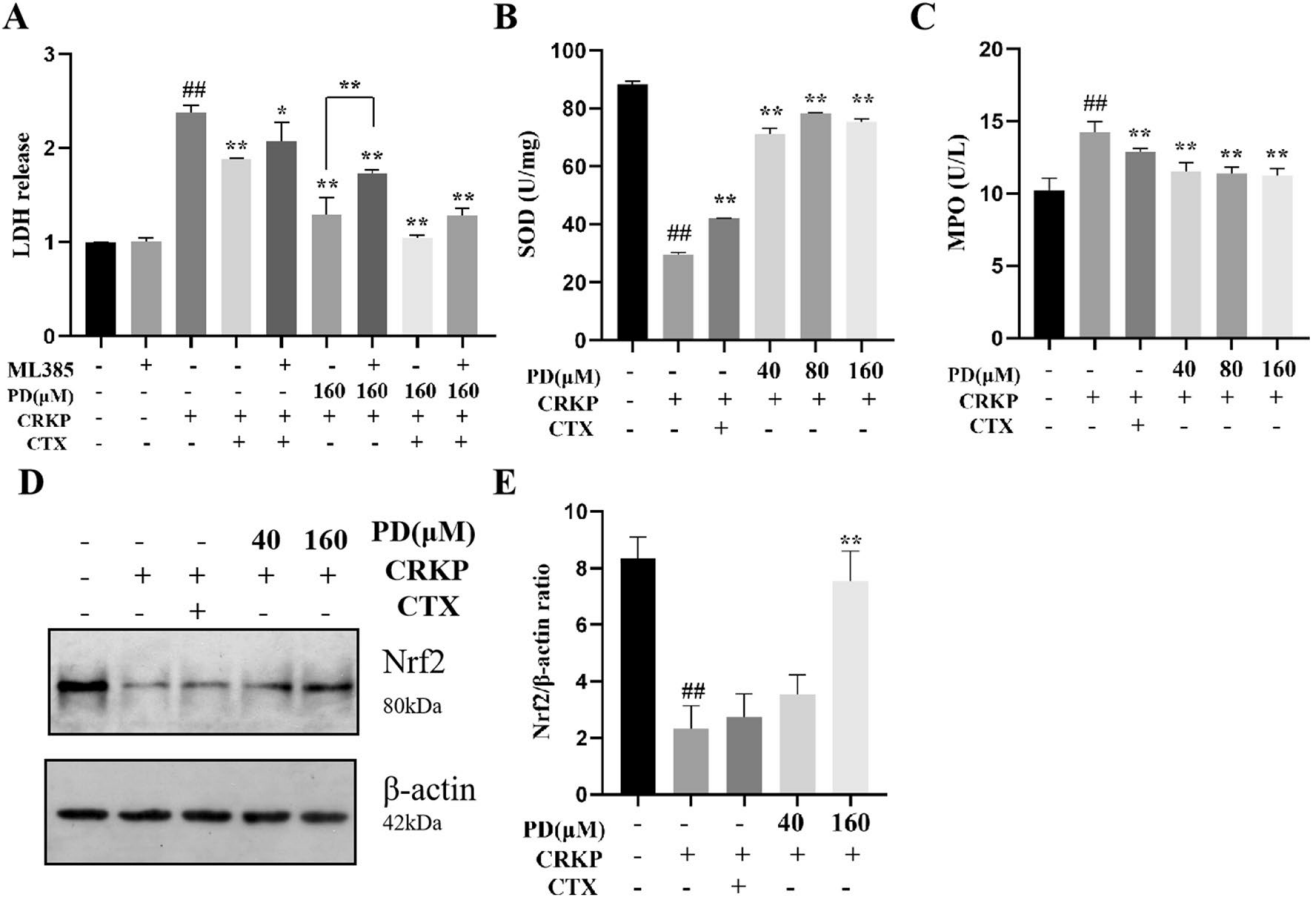
PD ameliorated oxidative stress by up-regulating the Nrf-2 signaling pathway in CRKP-infected A549 cells. CRKP-induced cellular injury induced by PD and ML385 was detected by LDH (**A**). The cells were incubated with CRKP and PD for 12 h and the concentration of SOD (**B**), MPO (**C**) were assessed by corresponding kits. A representative result of the protein expression of Nrf-2 among all groups was analyzed by western blot, and a statistical result was calculated (**D**-**E**). ^##^*P* < 0.01 vs. the control group; ^*^P < 0.05 and ^**^P < 0.01 vs. the CRKP alone group.

Nrf-2 was a transcription factor that coordinated the expression of cellular protective genes to maintain homeostasis^24^. In the above experiments, we know that PD could protect against CRKP-induced cell damage by activating Nrf-2 production. Next, we combined an Nrf-2 inhibitor (ML385) to further investigate the effect of phase on cell survival. First, the lung epithelial cells were pretreated with ML385 and then treated with PD and CRKP for 12 h. As shown in Fig. 8A, cell viability decreased with the ML385 combination compared to the group treated with PD alone. This result further indicated that PD could activate the Nrf-2 signaling pathway to promote cell survival. Activation of Nrf-2 activates the production of downstream antioxidant factors (Superoxide dismutase (SOD). As shown in Fig. 8B, compared with the control group, SOD was inhibited by CRKP infection, but PD interference reversed SOD production. In contrast, the CRKP-stimulated Myeloperoxidase (MPO), an oxidized protein, was produced in reduced amounts upon PD intervention (Fig. 8C). Here, we confirmed whether PD could activate the Nrf-2 pathway by WB analysis. As shown in Fig. 8D,E, the expression of Nrf-2 at the protein level was inhibited by CRKP, but its expression could be reversed by PD. In conclusion, PD could inhibit CRKP-induced oxidative stress by up-regulating Nrf-2.

## Discussion

Over the past few years, there have been affected by infections caused by enterobacteria, including the MDR and CRKP, which have been increasingly detected in healthcare facilities^25^. KP adopted different molecular strategies to develop resistance to carbapenem, including (1) production of carbapenemase (*blaKPC*, *blaOXA*, and metal-beta-lactamase); (2) nonexpression or mutation of *OmpK35* porin genes prevented bacteria acquisition of carbapenems through the outer membrane of carbapenems; and (3) up-regulation of the efflux system (*AcrB*), the latter two mechanisms often combined with high levels of other types of beta-lactamases^25,26^. Studies had shown that efflux pumps were associated with the formation of biofilms of pathogenic microorganisms, which was an important factor for the resistance of pathogenic microorganisms to antibiotics^27,28^. That could be attributed to the constitutive stress of antibiotics, which enhanced induced gene regulation and provided fitness advantages for resistant strains, leading to biofilm formation^29^. Resveratrol had shown its potential value in antibacterial therapy, which had been reported to enhance the antimicrobial efficacy of aminoglycosides against Staphylococcus aureus. PD, as the most abundant derivative of resveratrol in nature. Although the antioxidant and anti-inflammatory effects of PD had been well established, little was known about the nature of its antibacterial action^30^. At the same time, we found that PD had an inhibitory effect on biofilm and that the inhibitory effect on biofilm was greatly enhanced by combining with PD. PD exhibited antibacterial activity based on the killing time curve. Some studies had found that Rama overproduction and porin loss both decreased envelope permeability, and that porin loss had been demonstrated to raise carbapenem MICs for KP isolate-producing ESBLs or AmpC cephalosporinases, this was observed^31^. Proteomic analyses showed that this enhancement was mainly achieved by activating efflux pump production^32^. Therefore, we determined that PD could control AcrAB-TolC by regulating the regulatory factors *MarA*, *RamA*, *RobA,* and *SoxS* in AcrAB-TolC in CRKP, reducing the effects of CRKP efflux drugs. In addition, total bacterial protein concentrations were determined by SDS-PAGE. The results indicated that PD + CTX could reduce the total cellular protein content of CRKP, implying the expression of certain proteins in CRKP was suppressed by PD + CTX. In general, this research preliminarily clarified that PD might exhibit anti-CRKP activity by disrupting cell membrane integrity, affecting protein expression, and inhibiting AcrAB-TolC. However, the specific mechanism of action of PD on CRKP remained to be further studied in the future.

Oxidative stress was an imbalance between oxidative and antioxidant effects in the body that could lead to chronic inflammation, which activated multiple transcription factors, such as Nrf-2 and NF-κB/AP-1was an important node in the inflammatory response. Nrf-2 was an important transcription factor regulating oxidative stress, which up-regulated the production of many antioxidant proteins (such as SOD) once activated^33^. In the present study, we also investigated the beneficial effect of PD on the oxidative stress damage induced by CRKP in A549 cells. Mechanism studies revealed that PD increased the protein levels of Nrf-2 and up-regulated the protein levels of Cyto-c. In this study, we also found that PD reduced the content of MPO in lung epithelial cells after CRKP induction, and increased the activity of antioxidants such as SOD to combat oxidative stress response. This study speculated that PD might achieve a protective effect through the activation of the Nrf-2 pathway. In order to evaluate this possibility, we further adopted an Nrf-2 inhibitor to verify the role of Nrf-2 in CRKP-induced lung epithelial cells. We found that PD did not inhibit the oxidative stress of CRKP-induced lung epithelial cells inhibited by Nrf-2. These results suggested that Nrf-2 played a key role in the protective effect of PD on CRKP-induced lung epithelial cells. In addition, mitochondria were the main sites for the production of ROS, which were crucial in fighting infections; however, mitochondrial damage could lead to ROS increase, MMP changes, and morphological damage^34–36^. We measured ROS concentrations using flow cytometry and found a significant increase in CRKP response to cellular stimuli compared to controls. At the same time, PD exhibited antioxidant effects, which could be demonstrated by reducing ROS production. Meanwhile, compared with the control group, flow cytometry showed that MMP was significantly down-regulated after CRKP stimulation, while MMP down-regulated after PD treatment was improved. These results proved that PD could reduce CRKP-induced oxidative stress and inhibit the corresponding decline in MMP by regulating Nrf-2 levels.

At the same time, cytokines were required for immunity, and the initiation, continuation, and resolution of inflammation depended on a complex network of pro-inflammatory and anti-inflammatory cytokines; however, if their activity was overwhelming, it could cause sepsis, which could lead to multiple organ damage^37^. Therefore, controlling the production of proinflammatory cytokines was one of the strategies to treat infectious diseases. Previous clinical studies had reported that circulating levels of pro-inflammatory cytokines, including TNF-α, IL-6, and IL-8, were generally elevated in patients with pneumonia^38–41^. To further clarify the anti-inflammatory mechanism of PD, we used an ELISA kit to detect the expression levels of IL-1β, IL-6, TNF-α, and PEG2, and the results consistently showed that CRKP infection increased the levels of IL-1β, IL-6 and TNF-α in human lung epithelial cells. Moreover, the expression levels of IL-1β, IL-6, and TNF-α were decreased under the intervention of PD, compared with the CRKP group. PD reduced the expression of inflammatory cytokines during CRKP infection, but its anti-inflammatory effects need to be further verified.

In summary, our study was the first to report that the first antimicrobial effects of PD and PD alleviate CRKP-induced cell damage by activating the Nrf-2 signaling pathway. Given that PD had many outstanding advantages (low toxicity, no respiratory depression, and low addictive potential), it might become a new and interesting drug for the treatment of CRKP-induced bacterial pneumonia.

## Conclusion

**Figure 9:**
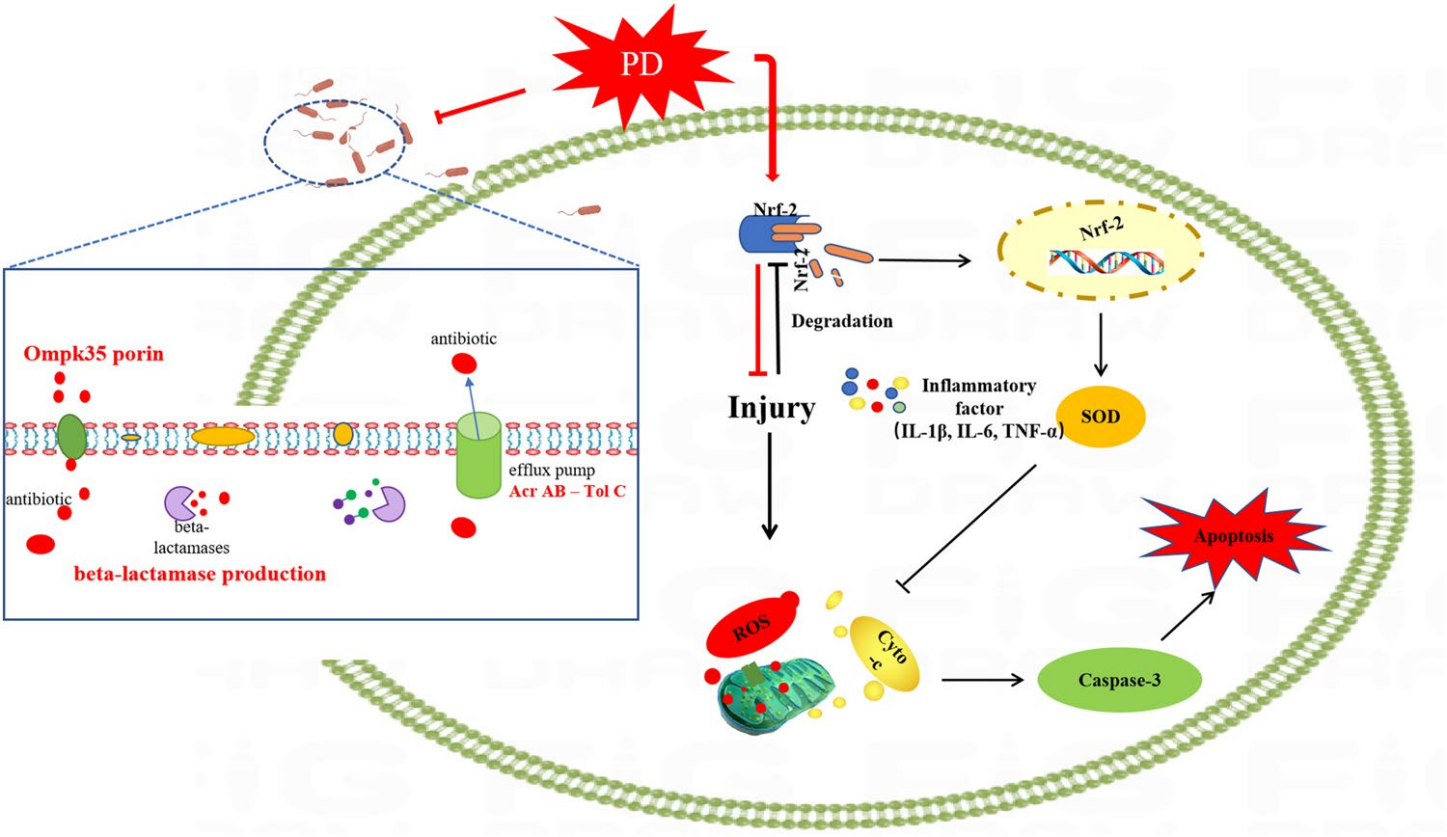
A schematic diagram showed that the antimicrobial mechanism of PD and the protection of mitochondrial integrity through the Nrf-2 pathway against the damage induced by CRKP in cells.

In the present study, PD showed significant antibacterial activity against CRKP by inhibiting the activity of the efflux pump AcrAB-TolC and biofilm integrity. Further, we identified an underlying intracellular mechanism by which PD triggered the activation of Nrf-2 to counteract the oxidative stress caused by CRKP, thus restoring dysfunctional mitochondria and reducing inflammation (Fig. 9). There were still limitations to the study that need to be considered. First, we showed that PD regulation of Nrf-2 exerts an antioxidant role in CRKP-induced cell damage in vitro. However, models of CRKP infection were not evaluated due to biosafety level limitations. Even though PD was able to suppress inflammation and oxidative stress in CRKP infection, the relationship between inflammation and antioxidants was not clear. Third, there were no animal experiments or primary experiments to evaluate the effect of PD on CRKP. We speculated that PD suppressed the secretion of inflammatory cytokines by reducing ROS, but its associated inflammatory signaling pathways should be further confirmed.

## Materials and methods

### Reagents and materials

PD was purchased from Chengdu Alfa Biotechnology Co., LTD. (HPLC ≥ 98%, Chengdu, China). The IL-1β, TNF-α, IL-6, and PGE2 ELISA kits were from Enzyme-labeled organisms (Jiangsu, China). The MPO, SOD were purchased from Nanjing Jiancheng Bioengineering Institute (Nanjing, China). The crystal violet was obtained from Beyotime (Shanghai, China).

### Bacteria culture

CRKP strain was isolated from Hangzhou First People's Hospital, and previous studies tested the resistance of CRKP to antibiotics^20^. CRKP strain was inoculated in LB broth under 200 rpm, 37 ℃.

### Crystal violet assay

The ability of biofilm formation was evaluated using the crystal violet method; specifically, bacterial suspensions of 10^8^ CFU/mL were added to a 96-well polystyrene microtiter plate. The absorbance at 595 nm was measured in accordance with the 0.1% crystal violet kit instructions. At the same time, the concentration of bacteria was observed with a scanning electron microscope.

### Gene expression testing by quantitative reverse transcription-PCR (qRT-PCR)

To detect the transcriptional expression levels of regulator genes RamA, MarA, RobA, and SoxS, as well as efflux pump genes *AcrA*, *AcrB*, and *TolC*, qRT-PCR was utilized. cDNA was prepared with ReverTra Ace® qPCR kit according to the instructions and prepared with SYBR® Green Realtime PCR Master Mix was amplified by QuantStudio 12 K Flex Real-Time PCR Syste. The relative expression of the gene was calculated by 2^−ΔΔCt^. The primer sequence is shown in Table 2.

**Table 2:** The primer sequences for qRT-PCR.

Gene	Forward (5′-3′)	Reverse (5′-3′)
*Acr A*	GGCAAACATGGATCAACTG	GGCGGTATCGTAGTCTTG
*Acr B*	GGAAGATACACCGCAGTT	TGTTAATGTCGCTGATGGA
*Tol C*	CTACGCTGTATAACGCTAA	CTAACGCCGACTTAATGT
*Mara*	ATGATGTCCAGACGTAATAATGA	GGCGATTCCAGGTTATCC
*Roba*	TATTCTATACCACCGCGCTGAC	GTGCCGTAGACGGTCAGGAT
*Soxs*	CTTAACATTGATATAGTCGCCAGA	CATCACGGTACGGAACATC
*KPC*	ATGTCACTGTATCGCCGTCT	TTTTCAGAGCCTTACTGCCC
*NDM*	GGTTTGGCGATCTGGTTTTC	CGGAATGGCTCATCACGATC
*OXA-48*	GCGTGGTTAAGGATGAACAC	CATCAAGTTCAACCCAACCG
*Rama*	GCTGCGTATTGATGATAT	TCTCCCTTGTACTGTAAA
*Ompk35*	GGATGGAAAGATGCCTTCAG	CATGACGAGGTTCCATTGT
*16sDNA*	AGGCCTAACACATGCAAGTC	GTGCAATATTCCCCACTGCTG

### SDS-PAGE analysis

Changes in protein expression were determined by sodium dodecyl sulfate–polyacrylamide gel electrophoresis (SDS-PAGE). All of the cellular proteins were denatured at 100 ºC for 10 min, then put through electrophoresis on a 5.0% stacked gel at 80 V for half an hour and a 10% resolving gel at 120 V for 70 min. After that, the gel was stained with Coomassie brilliant blue G250 until the protein bands were distinct. The image of the protein bands was obtained using the gel imaging system.

### Cell viability was determined by LDH, Annexin V and PI, and CCK8 assay

Culturing of A549 cells was conducted in F12K complete medium that comprised of 10% fetal bovine serum (FBS, GIBCO, Poncho) and 100 U/mL penicillin–streptomycin solution(Beyotime, Shanghai, China), at 37 ℃, 5% CO_2_.

CCK8 was used to assess cell viability. Seeding A549 cells in 96-well plates for 24 h, cells were exposed to different concentrations of PD (20, 40, 80, or 160 μM), then 10 μL of CCK8 was added to each well for 1–2 h. Cell viability was assessed by absorbance (optical density) with a microplate reader at 450 nm. The effect of PD on CRKP-induced cells was determined by the LDH assay. The absorbance at 490 nm was measured using a microplate reader, according to the LDH kit (Source leaf, Shanghai, China). Apoptosis was detected by using the FITC Annexin V Apoptosis Assay kit (BD, Guangzhou, China). The role of Nrf-2 in CRKP-induced cell damage was further investigated using Nrf-2 inhibitors (ML385). The experiments were tested with the LDH kit.

### ROS and MMP were measured by flow cytometry

Intracellular ROS levels as a general indicator of oxidative stress were determined using a dichloro-dihydro-fluorescein diacetate (DCFH-DA) probe. MMP was evaluated using a fluorescent indicator JC-1 kit (Beyotime, Shanghai, China).

### Determination of oxidative protein/antioxidant protein and inflammatory cytokines

The contents of MPO, SOD in CRKP-infected cells were analyzed. MPO activity was determined using an MPO assay kit. SOD activity in cell lysates was measured using SOD kits, respectively.

The cells were treated with CRKP and PD for 12 h. The concentrations of TNF-α, IL-6, IL-1β, and PGE2 in cell-free supernatant were determined by the quantitative factor ELISA ki.

### Adhesion assay and invasion assay

For the adhesion experiment, after 12 h treatment, each group was washed 3 times with PBS and soaked in 0.25% pancreatic enzyme100 μL and 0.5% TritonX-400 μL for 10 min. Then, 100 μL of the diluted solution was placed on LB medium and incubated at 37℃. Finally, the cells were counted and analyzed. In the invasion experiment, infected cells were treated as described above for 12 h, and 500 μL of tigecycline (TIG) cell basal medium was added to each group to kill extracellular bacteria, washed in PBS, and treated according to steps in the adhesion test.

### Western blot

Protein concentration was determined by using a BCA kit. Then, the same amount of protein was electrophoresed in 12% polyacrylamide gel, transferred to the PVDF membrane, and sealed with 5% fat-free milk powder for 1 h. The membranes were then incubated with rabbit anti-human β-actin (Abcam, China), Nrf-2 (Abcam, China), and Cyto-c (CST, China) antibodies at 4 ℃ overnight. Tris–HCl-Tween (TBST) was used for 10 min and incubated with an anti-rabbit IgG secondary antibody (ThermoPierce, China) at room temperature for 1 h. Ultimately, the gel Imager (Bio-Red, Shanghai, China) was used to visualize the membrane using an enhanced chemiluminescence (ECL) agent. The results were analyzed by ImageJ software. β-actin was used as endogenous control and normalization.

### Statistical analysis

All data were expressed as mean ± standard deviation (SD). Differences between the groups were followed by a one-way ANOVA multiple comparison test. There were significant differences at P < 0.05 (significant) or P < 0.01 (highly significant).

### Ethics approval

The *carbapenem-resistant* *Klebsiella pneumoniae* (CRKP) strain was reviewed and approved by the Hangzhou First People's Hospital ethics committee, Hangzhou, China. We confirmed that informed consent was obtained from the study participants. We confirmation that the guidelines outlined in the Declaration of Helsinki were followed.

### Supplementary Information

Supplementary Information 1.

Supplementary Information 2.

Supplementary Information 3.

## Data Availability

All data generated or analyzed during this study are included in the Supplementary Information files.

## Abbreviations

KP: *Klebsiella pneumoniae*
CRKP: *Carbapenem-resistant* *Klebsiella pneumoniae*
PD: Polydatin
ROS: Reactive oxygen species
YHPG: Yinhuapinggan
Nrf-2: Nuclear factor erythroid 2-related factor 2
CTX: Cefotaxime sodium
SEM: Scanning electron microscope
MMP: Mitochondrial membrane potential
IL-1β: Interleukin-1β
TNF-α: Tumor necrosis factor-α
IL-6: Interleukin-6
PGE2: Prostaglandin E2
ELISA: Enzyme-linked immunosorbent assay
MPO: Myeloperoxidase
SOD: Superoxide dismutase
TIG: Tigecycline
TBST: Tris–HCl-tween
ECL: Enhanced chemiluminescence
SD: Standard deviation
