# Supplementary figures and images for: Polydatin prevent lung epithelial cell from Carbapenem-resistant Klebsiella pneumoniae injury by inhibiting biofilm formation and oxidative stress
Source: Sci Rep. 2023 Oct 18;13:17736. doi: 10.1038/s41598-023-44836-7 (PMC10584862; doi:10.1038/s41598-023-44836-7)

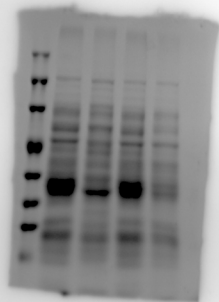

Supplement: Supplementary file 1 — Supplementary Information 1. [file 41598_2023_44836_MOESM1_ESM.pdf]

Cyto-c raw data:

|   |   |   |    |     |              |
|---|---|---|----|-----|--------------|
| - | - | - | 40 | 160 | PD( $\mu$ M) |
| - | + | + | +  | +   | CRKP         |
| - | - | + | -  | -   | CTX          |

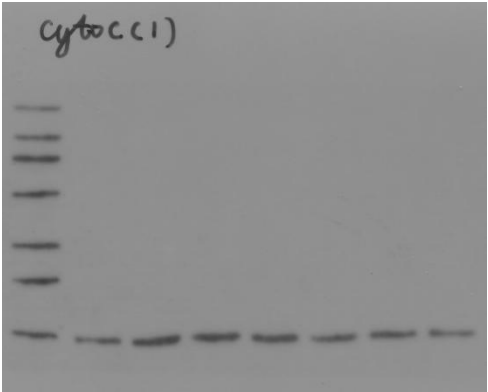

$\beta$ -actin raw data:

|   |   |   |    |     |              |
|---|---|---|----|-----|--------------|
| - | - | - | 40 | 160 | PD( $\mu$ M) |
| - | + | + | +  | +   | CRKP         |
| - | - | + | -  | -   | CTX          |

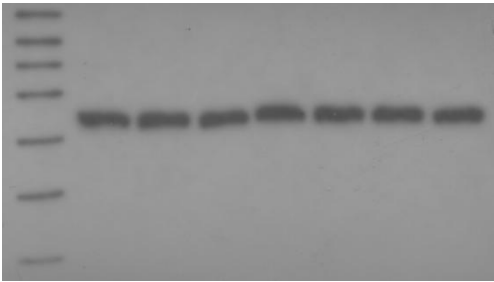

Supplement: Supplementary file 2 — Supplementary Information 2. [file 41598_2023_44836_MOESM2_ESM.pdf]

Nrf-2 raw data:

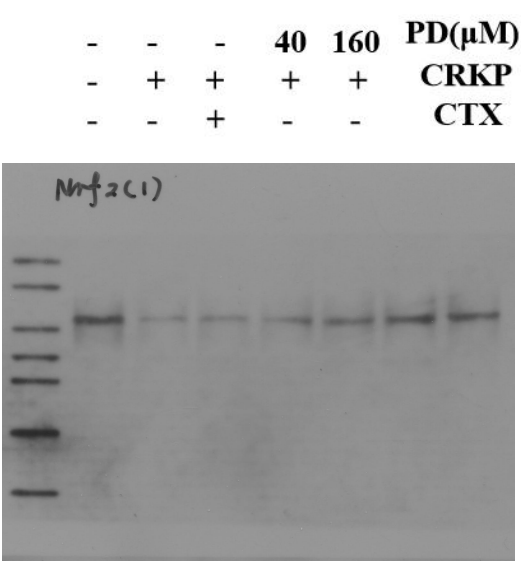

$\beta$ -actin raw data:

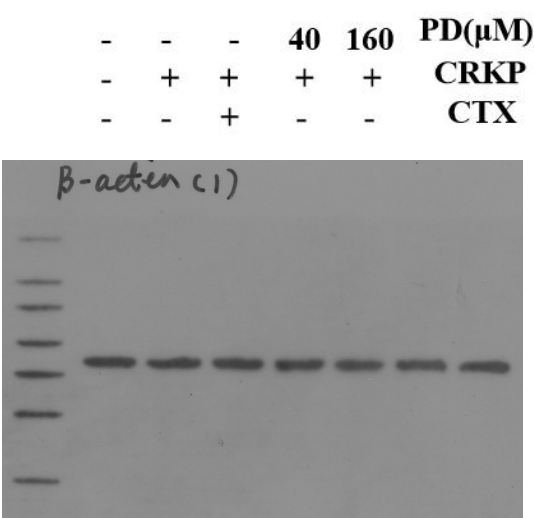

Supplement: Supplementary file 3 — Supplementary Information 3. [file 41598_2023_44836_MOESM3_ESM.pdf]
